# First clinical experience of correcting phantom‐based image distortion related to gantry position on a 0.35T MR‐Linac

**DOI:** 10.1002/acm2.13404

**Published:** 2021-10-06

**Authors:** Benjamin C. Lewis, Jaeik Shin, Benjamin Quinn, Enzo Barberi, Domenic Sievert, Jin Sung Kim, Taeho Kim

**Affiliations:** ^1^ Department of Radiation Oncology Washington University School of Medicine St. Louis, Missouri USA; ^2^ Department of Radiation Oncology Yonsei University College of Medicine Seoul Republic of Korea; ^3^ Department for the Modus Medical Modus Medical Devices Inc. London Ontario Canada

**Keywords:** distortion correction, MR‐Linac, MRgRT

## Abstract

MR‐guided radiotherapy requires strong imaging spatial integrity to deliver high quality plans and provide accurate dose calculation. The MRI system, however, can be compromised by the integrated linear accelerator (Linac), resulting in inaccurate imaging isocenter position and geometric distortion. Dependence on gantry position further complicates the correction of distortions. This work presents a new clinical application of a commercial phantom and software system that quantifies isocenter alignment and geometric distortion, as well as providing a deformation vector field (DVF). A large distortion phantom and a smaller grid phantom were imaged at multiple gantry angles from 0 to 330° on a 0.35 T integrated MR‐Linac. The software package was used to assess geometric distortion and generate DVFs to correct distortions within the phantom volume. The DVFs were applied to the grid phantom with resampling software then evaluated using structural similarity index measure (SSIM). Scans were also performed with a ferromagnetic clip near the phantom to investigate the correction of more severe artifacts. The mean magnitude isocenter shift was 0.67 mm, ranging from 0.25 to 1.04 mm across all angles. The DVF had a mean component value of 0.27 ± 0.02, 0.24 ± 0.01, and 0.19 ± 0.01 mm in the right‐left (RL), anterior‐posterior (AP), and superior‐inferior (SI) directions. The ferromagnetic clip increased isocenter position error from 1.98 mm to 2.20 mm and increased mean DVF component values in the RL and AP directions. The resampled grid phantom had an increased SSIM for all gantry angles compared to original images, increasing from 0.26 ± 0.001 to 0.70 ± 0.004. Through this clinical assessment, we were able to correct geometric distortion and isocenter shift related to gantry position on a 0.35 T MR‐Linac using the distortion phantom and software package. This provides encouragement that it could be used for quality assurance and clinically to correct systematic distortion caused by imaging at different gantry angles.

## 1 INTRODUCTION

Magnetic resonance guided radiotherapy (MRgRT) has become an important treatment modality for improving treatment outcomes by increasing local control rates and reducing normal tissue toxicity.^1^, ^2^, ^3^ Systems that combine a magnetic resonance imaging (MRI) scanner and a linear accelerator (Linac) provide beam gating, real‐time target tracking, and the ability to perform online adaptive radiotherapy.^3^, ^4^, ^5^ Additionally, soft tissue contrast and structure identification with on‐board MRI systems are superior to that provided by on‐board X‐ray systems, both for 2D planar images and volumetric images such as cone beam CT (CBCT), without the addition of any excess ionizing radiation.^6^, ^7^

Every advantage provided by MRI guidance is dependent on the spatial and geometric fidelity of the resulting images. MR images have intrinsic distortions which must be addressed prior to using images for target tracking or adaptive planning to prevent inaccurate dose calculation or improper beam gating.^8^, ^9^, ^10^ These intrinsic distortions are generated by gradient nonlinearity, main static magnetic field (B0) inhomogeneity, imperfect shimming, and eddy currents, and manifest as distorted patient surface, spatial shift of the imaging volume, and distortion of internal structures.^8^, ^11^, ^12^, ^13^, ^14^ On an MR‐Linac system additional sources of distortion are introduced by the radiotherapy system due to imperfect radiofrequency shielding for Linac components and the presence of ferromagnetic material around the MRI bore. Hu et al. provided a comprehensive QA program for characterizing imaging performance on the Cobalt‐60 based ViewRay MRgRT system.^15^ They measured spatial integrity at a single gantry angle and found an average geometric distortion of 1.5 and 2.7 mm at 20 and 35 cm diameter spherical volumes (DSV), respectively, when compared to CT marker positions. Additionally, magnetic field homogeneity at five gantry angles was measured, showing that over a 45 cm, DSV the peak‐to‐peak variation was less than 25 ppm at all measured angles. Additional studies at multiple gantry angles have shown that the position of the Linac gantry can impact the imaging isocenter position by nearly 2 mm and deform rigid bodies over the entire field of view.^16^, ^17^, ^18^ For clinical imaging with the ViewRay MR‐Linac system the manufacturer recommends using a single home gantry position for acquiring all setup and planning images, where the imaging system is tuned at commissioning. Comprehensive MRgRT QA protocols have been developed on other systems, such as the 1.5 T Elekta Unity MR‐Linac system.^19^ The work by Tijssen et al. showed a varying level of gantry angle dependency for B0 field homogeneity across four Elekta Unity systems and four gantry angles, with one system showing no dependency and another displaying increased inhomogeneity with active shimming in place.

We previously reported the impact of gantry position on image quality for the ViewRay MRIdian 0.35 T MR‐Linac system (Oakwood Village, OH) using a Fluke 76‐907 uniformity and linearity phantom (HP Manufacturing, Cleveland, OH), a proprietary software provided by ViewRay for spatial integrity analysis, and imaging sequences provided by ViewRay. While this study produced valuable information, it relied on assessing machine performance with software provided by the same company. Additional studies have been performed using in‐house volumetric phantoms to assess distortion on low‐field MR images at multiple radiotherapy centers, for a limited number of gantry angles.^20^, ^21^, ^22^ However, the in‐house phantoms have been heavy and complicated to properly set up. A lightweight spatial integrity phantom and analysis software produced by Modus Medical Devices Inc. (Modus QA) (London, Ontario, Canada) offers the possibility to examine the imaging distortion with a third‐party system. In addition to assessing image distortion, the phantom can be used to generate deformation vector fields (DVFs) to correct systematic distortions over a large field of view.

This study presents the first clinical experience with the Modus QA QUASAR™ MRID^3D^ geometric distortion phantom and software system to measure the spatial distortion and imaging isocenter shift related to gantry position on an MR‐Linac system. Using a deformation vector field generated by the software system from the MRID^3D^ geometric distortion phantom, we also corrected the distorted image back to the original geometry, providing the possibility for systematic distortion correction of MR images. DVFs were also applied to an independent phantom at different gantry angles and to the MRID^3D^ geometric distortion phantom in the presence of metal artifact distortion to assess systematic corrections.

## 2 METHODS

### 2.1 Spatial integrity phantoms

**FIGURE 1:**
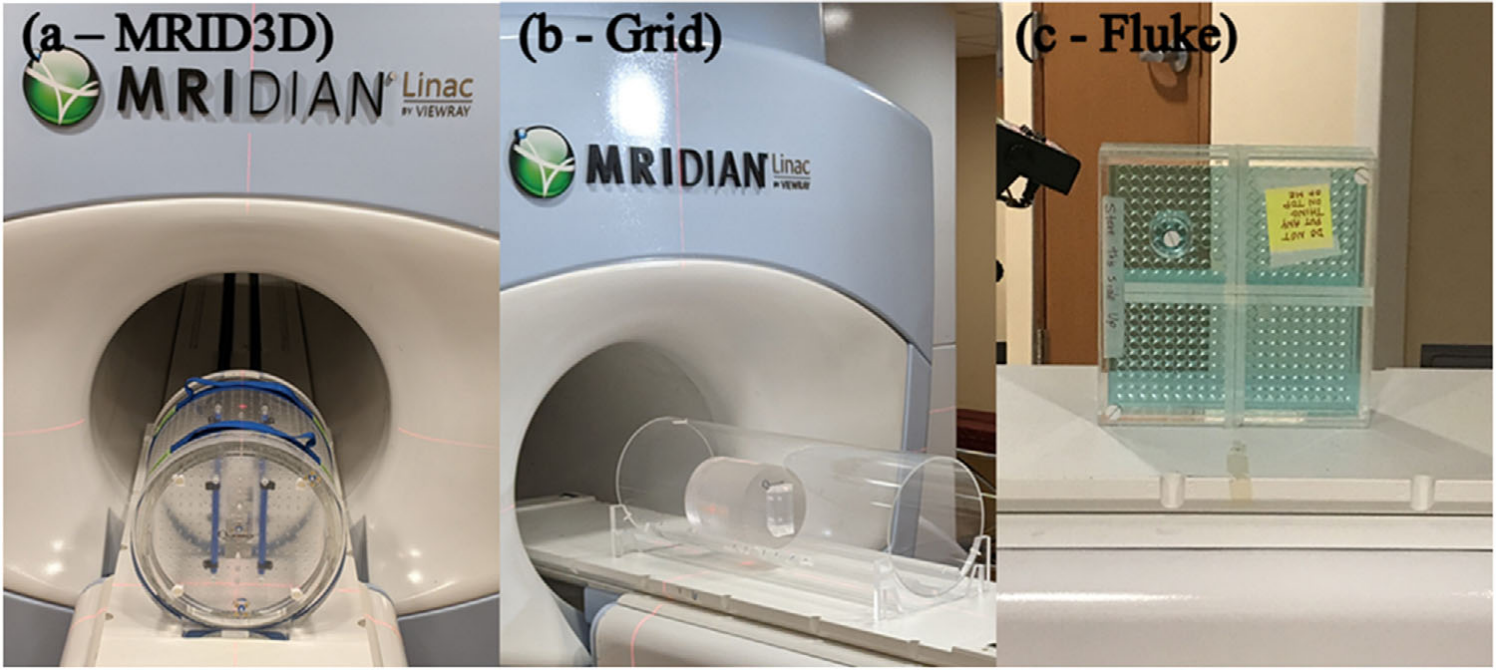
Images of the (a) QUASAR^TM^ MRID^3D^, (b) Modus QA grid validation phantom, and (c) the Fluke 76‐907 Uniformity and Linearity phantoms are shown during setup on the 0.35T MR‐Linac

**FIGURE 2:**
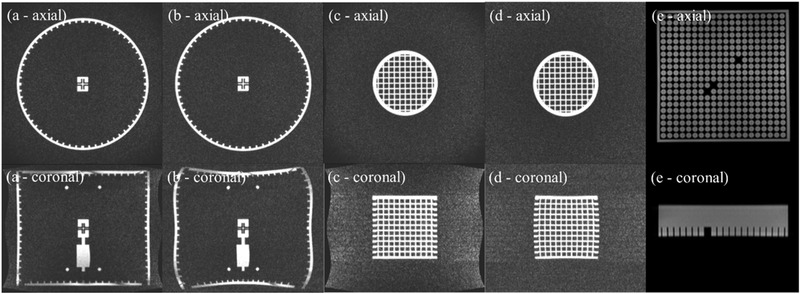
Axial and coronal views of all phantoms referenced in this study. The QUASAR^TM^ MRID^3D^ cylindrical phantom is shown with the on‐board distortion correction turned on (a) and off (b). The Modus QA grid validation phantom is shown with the on‐board distortion correction turned on (c) and off (d). The Fluke 76‐907 Uniformity and Linearity phantom that was used in previous studies

This study used two phantoms that were both imaged on a 0.35 T ViewRay MRIdian MR‐Linac system in MRI QA mode. 3D slicewise images were acquired with the gantry in 12 different positions from 0° to 330° in 30° increments. The first phantom imaged was the QUASAR™ MRID^3D^ cylindrical phantom (Modus QA, London, Ontario, Canada). This phantom has a diameter of 394 mm and a length of 391 mm. The phantom interior is a 25 L air‐filled space, with a closed surface containing 1502 machined fiducials containing paraffinic mineral oil. Fiducials are 6 mm long and have a diameter of 3 mm, with the exception of six positioning fiducials that are 5 mm in diameter. All fiducials are spaced at 18 mm increments. This acquisition was repeated for the MRID^3D^ cylindrical phantom over two separate sessions (S1 and S2). The second phantom was a 3D grid validation phantom provided by Modus QA. This cylindrical phantom incorporates regularly spaced acrylic grids into the central mineral oil‐filled volume. The grid portion of the phantom is 150 mm across and 150 mm long, with each grid voxel containing a volume of 15 × 15 × 15 mm^3^. A phantom holder that was built in‐house was used to hold the phantom in place. Both phantoms are shown in Figure 1. The same clinical TRUFI sequence was used for each phantom with TR/TE: 3.0/1.0 ms, flip angle: 60°, FoV: 450 × 450 × 360 mm^3^, acquisition voxel size: 1.5 × 1.5 × 1.5 mm^3^, readout BW: 570 Hz/Px. Images were also acquired with the on‐board scanner distortion correction function turned on (DstOn) and off (DstOff) and the phase encoding direction applied in both the anterior‐posterior (AP) and posterior‐anterior directions, according to manufacturer instruction. Figure 2 displays axial and coronal views of the QUASAR™ MRID^3D^ cylindrical and 3D grid validation phantoms with DstOn and DstOff.

The geometric distortion and isocenter shift values were compared to previous work by this group in which they were measured for multiple gantry angles using a 2D spatial integrity phantom, the 2D Fluke 76‐907 Uniformity, and Linearity water phantom. The 2D spatial integrity phantom contains a plane of marker posts and must be setup in at least three orientations to acquire sufficient information. This phantom is used along with the Spatial Integrity Analysis 2D software provided by ViewRay for quality assurance. The phantom is shown in Figure 1, and MR images in the axial and coronal planes are shown in Figure 2.

An additional set of distortion scans was completed at a gantry angle of 300°. For these images, a 33 mm long ferromagnetic clip (i.e., paperclip) was placed in a plastic container and bound with paper packing material. The plastic container was then secured to the treatment table in the same axial plane as the imaging isocenter using tape. The container was in the same position for each phantom and the treatment table was returned to the same absolute position readout after switching between phantoms. The same scan parameters were used for these acquisitions, with the ferromagnetic clip in place and removed from the room, and the distortion correction was also set to on and off.

### 2.2 Geometric distortion analysis and correction

MRID^3D^ phantom images were uploaded to the QUASAR™ MRID^3D^ geometric distortion analysis software. The software produced principal component error values based on harmonic analysis of the fiducials as well as imaging isocenter alignment. All isocenter shifts are relative to gantry angle of 300°. geometric distortion and isocenter shift values were recorded for the entire phantom. DVFs were then exported for all scans. The exported DVFs were applied to the corresponding 3D grid validation phantom images using prototype System distortion resampler software provided by Modus QA. Resampled images were then compared to the original and resampled images acquired at gantry angle of 0° where the system baseline shimming was set during a system upgrade. Structural similarity index measure (SSIM) was calculated in MATLAB 2020b (MathWorks, Natick, MA).

## 3 RESULTS

### 3.1 Gantry dependent geometric distortion

**FIGURE 3:**
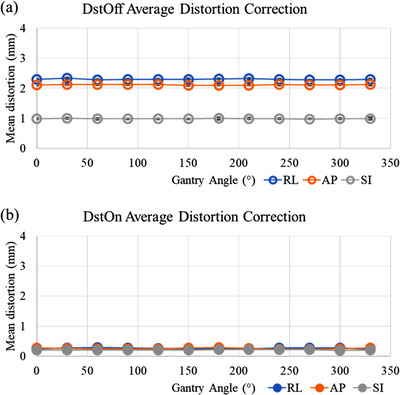
Mean distortion correction values for the right‐left (RL), anterior‐posterior (AP), and superior‐inferior (SI) directions across all gantry angles for imaging sessions 1 and 2, with the phase encoding direction in the anterior‐posterior (AP) and posterior‐anterior (PA) directions. Plots show the values with (a) distortion correction off (DstOff) and with (b) distortion correction on (DstOn)

Volumetric 3D images were acquired in one orientation for the QUASAR™ MRID^3D^ cylindrical phantom due to its large field of view. Figure 3 shows the mean (±1 SD) DVF components by gantry angle for images acquired with DstOff (Figure 3a) and DstOn (Figure 3b). Average DVF values at gantry angle of 300° from the two imaging sessions for DstOff scans were 2.12 ± 0.01, 0.99 ± 0.02, and 2.29 ± 0.01 mm in the anterior‐posterior (AP), superior‐inferior (SI), and right‐left (RL) directions, respectively. Average DVF values at gantry angle of 300° from the two imaging sessions for DstOn scans were 0.24 ± 0.01, 0.19 ± 0.01, and 0.27 ± 0.02 mm in the AP, SI, and RL directions, respectively. The maximum DVF values at gantry angle of 300° for the DstOff scans were 15.26, 5.71, and 15.32 mm, in the AP, SI, and RL directions, respectively, and were 1.61, 1.46, and 1.40 mm in the AP, SI, and RL directions for the DstOn scans.

**FIGURE 4:**
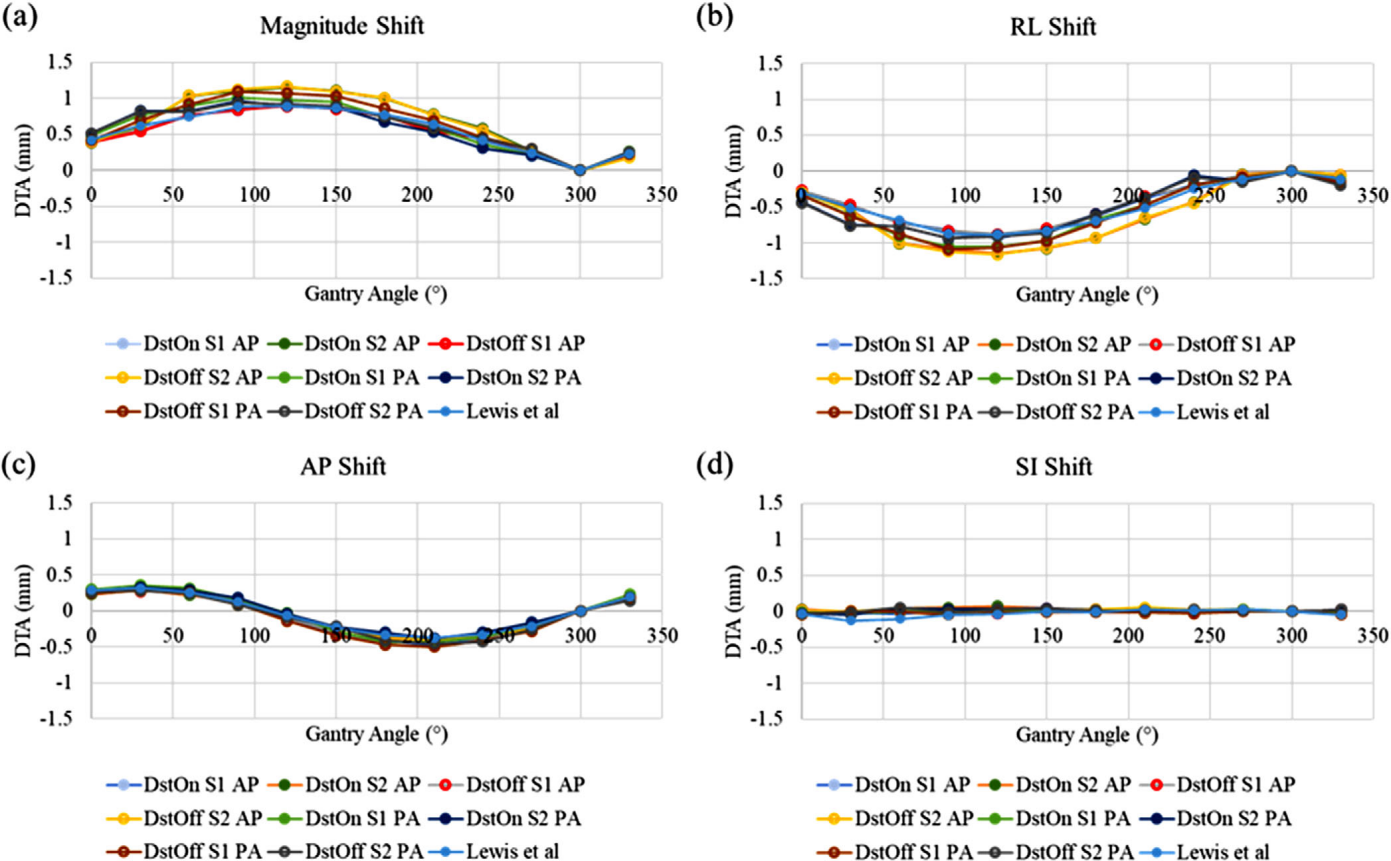
Isocenter distance to agreement (DTA) for the (a) magnitude, (b) right‐left (RL), (c) anterior‐posterior (AP), and (d) superior‐inferior (SI) directions. Each plot includes the values from both imaging sessions with the phase encoding direction in the anterior‐posterior (AP) and posterior‐anterior (PA) directions, and with the distortion correction turned on (DstOn) and off (DstOff), as well as the previous data from Lewis et al^18^

Isocenter shift values for each gantry angle are shown in Figure 4, in the AP, SI, and RL directions, as well as the magnitude shift value. The maximum magnitude isocenter shift of 1.04 and 1.02 mm for DstOn and DstOff, respectively, occurred at a gantry angle of 120° for the AP phase encoding direction. The PA phase encoding direction had a maximum magnitude isocenter shift of 1.01 and 1.02 mm for DstOn and DstOff, respectively, at a gantry angle of 90°.

In order to assess the precision of the isocenter shift measurements, the system distortion resampler software was used to generate copies of an AP phase encoding direction DstOn scan, shifted in the y‐direction by amounts ranging from 0.05 to 1.5 mm. The detected centroid values from these images were compared to the expected position, which is the detected position in the original image plus the applied shift. The resulting measurement error ranged from −0.05 to 0.09 mm. A comparison of sets of four series acquired on the same day and at the same gantry angle showed that the maximum variation in the in‐plane phase encoding component of the centroid within each group was 0.06 mm.

### 3.2 Distortion correction

#### 3.2.1 Gantry position

**FIGURE 5:**
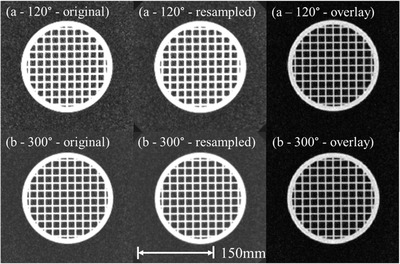
The grid phantom acquired at gantry angle 120° (a) and 300° (b), showing the original images and the resampled images using the appropriate deformation vector field generated using the QUASAR^TM^ MRID^3D^ geometric distortion analysis software, and an overlayed image showing matching pixels in grayscale. The scale bar indicates the 150 mm length

Application of the DVFs from the MRID^3D^ cylindrical phantom distortion measurement was successfully applied to the grid phantom for resampling. Figure 5 shows the grid phantom pre‐ and post‐correction for two gantry angles (120° and 300°). The field of view is cut off due to the resampler only applying the DVF to the volume encompassed by the MRID^3D^ cylindrical phantom.

**FIGURE 6:**
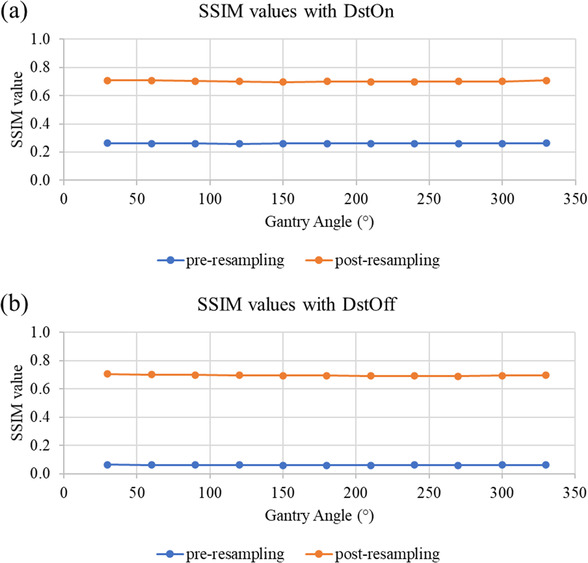
SSIM values for all measured gantry angles pre‐ and post‐resampling. Shown with the onboard distortion correction function turned on (a) and turned off (b)

Resampling increased the average SSIM value for all gantry angles relative to resampled gantry 0° images from 0.26 ± 0.001 to 0.06 ± 0.00 prior to resampling, from 0.70 ± 0.004 to 0.69 ± 0.005 post resampling for DstOn and DstOff, respectively. SSIM was evaluated against the resampled gantry 0° image due to slight blurring of structures after resampling. Figure 6 displays SSIM values for DstOn and DstOff images at all gantry angles, pre‐ and post‐resampling.

#### 3.2.2 Metallic object in‐field

**FIGURE 7:**
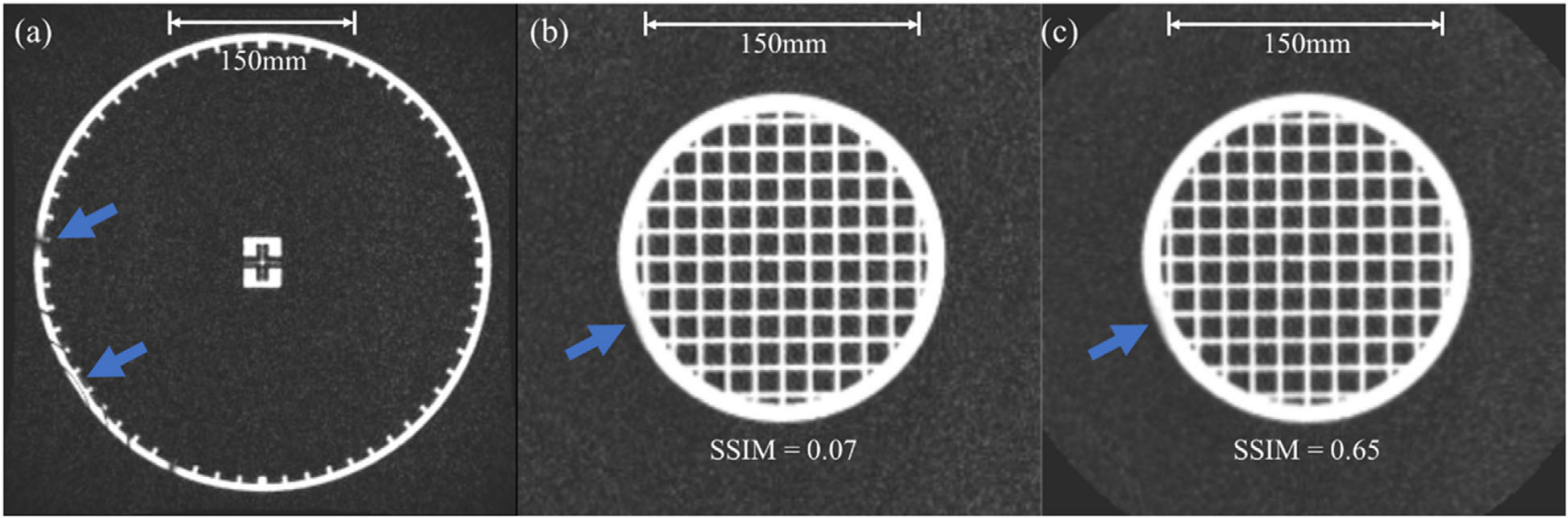
The QUASAR^TM^ MRID^3D^ phantom (a), Modus QA grid validation phantom pre‐resampling (b), and the Modus QA grid phantom post‐resampling (c) with a ferromagnetic clip in place on the treatment table in the same axial plane as the image. The blue arrows indicate regions of distortion caused by the ferromagnetic clip in all three images. The scale bar indicates a 150 mm length for each image

With the ferromagnetic clip in place, the geometric distortion of the MRID^3D^ cylindrical phantom was increased from 0.27 ± 0.31 to 0.36 ± 0.44 mm in the RL direction and from 0.24 ± 0.34 to 0.27 ± 0.36 mm in AP direction. The correction remained constant in the SI direction at 0.29 ± 0.33 mm. The imaging isocenter distance to agreement increased from a magnitude of 1.98 to 2.20 mm with the paperclip in place. Slight distortions were visible near the edge of the field. The DVF was then applied to the grid phantom using the system distortion resampler software. The SSIM value between the resampled grid phantom without the ferromagnetic clip and the grid phantom with the ferromagnetic clip was 0.07 pre‐resampling and was 0.65 post‐resampling. The QUASAR™ MRID^3D^ cylindrical phantom, and pre‐ and post‐correction grid phantom images with the paperclip in place are displayed in Figure 7.

## 4 DISCUSSION

This study presents the first clinical experience with the gantry‐related image distortion correction using the QUASAR™ MRID^3D^ cylindrical phantom and the associated geometric distortion analysis software on an institutional 0.35 T MR‐Linac system. This phantom‐based approach to measure geometric distortion and isocenter shift allowed for a vendor independent alternative to the current clinical practice that utilizes software provided by ViewRay to perform quality assurance on a ViewRay machine. This system also uses a large 3D phantom which covers a large imaging field of view. Measuring distortion over a large field of view is valuable for assessing distortion near the periphery of the imaging region without the need for repositioning the phantom and acquiring additional image sets. The geometric distortion and isocenter shift were quantified at multiple gantry angles, additionally, large field of view DVFs were produced to correct the systematic distortions present in acquired MR images. Comparison of isocenter shifts to previous data published by Lewis et al.,^18^ which used the Fluke spatial integrity phantom, showed good agreement with the maximum isocenter shift of 0.89 mm occurring at gantry angle of 120°, as it did in this work. The calculated DVF allowed for an independent smaller grid phantom to be corrected back to its original geometry over multiple gantry angles. This gives the possibility for gantry angle‐specific systematic distortion corrections to be applied to clinical images. Geometric correction of clinical images at multiple gantry angles could reduce imaging time and the need for a set gantry imaging position when re‐acquisition of 3D anatomical images is required during treatment. Additionally, the software was able to correct for a small metal artifact created by a paperclip at the edge of the field. This indicates that if different systematic distortions are present on different imaging systems, that the software could correct them. However, it cannot be used to correct for a patient‐specific metallic artifact because it requires a reference template.

MRI isocenter shift results showed that there is a systematic change in isocenter position with gantry angle. The results in this study closely agree with previous works which used different phantoms and analysis techniques.^16^, ^18^ This work also found that the on‐board distortion correction was able to correct the geometric distortion to a similar value at all gantry angles, and was a significant improvement over images with the distortion correction turned off. Gantry angle of 0° showed a higher distortion correction value in the AP direction for DstOn scans than other gantry angles, but was the distortion was still corrected by the software.

Future work with this system will be directed at systematic distortion correction of human images acquired at multiple gantry angles. However, this extension presents many challenges, including assessing correction accuracy and the long acquisition time for imaging at multiple gantry angles. The phantom provides a large field of view, but it may not be sufficient to capture a human subject which could result in an incomplete distortion correction.

This work demonstrated the ability to perform phantom‐based image distortion correction via quantifying and applying deformation vectors to a large cylindrical phantom in a clinical setting and provided a reliable alternative to current imaging quality assurance procedures on integrated MR‐Linac systems.

## 5 CONCLUSION

The first clinical experience with a new geometric distortion analysis and correction system was presented in this work. This system showed good agreement in imaging isocenter and image distortion values as seen in previous work, with imaging isocenter position varying depending on gantry angle and having a maximum deviation of 1.04 mm, with the distortion correction on. Large volume DVFs could also be applied to correct systematic geometric distortion in a secondary phantom, even in the presence of metal‐induced artifacts.

## CONFLICT OF INTEREST

The authors declare no conflicts of interest in this work.

## AUTHOR CONTRIBUTIONS

Benjamin Lewis contributed to data collection, analysis, and wrote the manuscript. Jaeik Shin contributed to data collection. Benjamin Quinn and Enzo Barberi contributed to design of data analysis, analysis tools, and provided technical assistance. Domenic Sievert contributed to data collection and analysis. Jin Sung Kim contributed to study design and supervision. Taeho Kim contributed to data collection, analysis, and supervision of the project. All authors discussed the results and contributed to the final manuscript.

## Data Availability

The data that support the findings of this study are available from the corresponding author upon reasonable request.
